# The Aging Network and the Older Americans Act Reauthorization

**DOI:** 10.1093/geroni/igaa057.2572

**Published:** 2020-12-16

**Authors:** Amy Gotwals

**Affiliations:** National Association of Area Agencies on Aging, Washington, District of Columbia, United States

## Abstract

This presentation will cover a range of OAA reauthorization proposals - successes and what was not possible this year. As a leader in the aging network, n4a played a major role in the legislative process, and the discussion will include top priorities for improving the ability of the network to serve older adults with community-based supports and services.

